# Regulation of Inflammatory Cell Death by Phosphorylation

**DOI:** 10.3389/fimmu.2022.851169

**Published:** 2022-03-01

**Authors:** Wen Xu, Yi Huang

**Affiliations:** ^1^ Neurology Department, The First Affiliated Hospital of USTC, Division of Life Sciences and Medicine, University of Science and Technology of China, Hefei, China; ^2^ Wuxi School of Medicine, Jiangnan University, Wuxi, China

**Keywords:** phosphorylation, pyroptosis, apoptosis, necroptosis, ferroptosis

## Abstract

Cell death is a necessary event in multi-cellular organisms to maintain homeostasis by eliminating unrequired or damaged cells. Currently, there are many forms of cell death, and several of them, such as necroptosis, pyroptosis and ferroptosis, even apoptosis trigger an inflammatory response by releasing damage-associated molecular patterns (DAMPs), which are involved in the pathogenesis of a variety of human inflammatory diseases, including autoimmunity disease, diabetes, Alzheimer’s disease and cancer. Therefore, the occurrence of inflammatory cell death must be strictly regulated. Recently, increasing studies suggest that phosphorylation plays a critical role in inflammatory cell death. In this review, we will summarize current knowledge of the regulatory role of phosphorylation in inflammatory cell death and also discuss the promising treatment strategy for inflammatory diseases by targeting related protein kinases that mediate phosphorylation or phosphatases that mediate dephosphorylation.

## Introduction

Cell death is a process common to all creatures. Early classified cell death based on morphology into apoptosis, autophagy, and necrosis. According to the latest recommendations by the Nomenclature Committee on Cell Death (NCCD) in 2018, there are two types of cell death named programmed cell death (PCD) and non-PCD (1, 2). Non-PCD is referred as accidental cell death (ACD), Which is stimulated by many external factors, such as infection, toxins and physical damage that cause morphological changes, for instance cytoplasmic swelling, rupture of the plasma membrane, and subsequent loss of cell contents, there is no severe chromatin condensation, but with random degradation of DNA (3). ACD is common in ischemia, tumor, and some form of neurodegeneration disease. It is often thought of as a passive process (4, 5). PCD can be regulated by drug or genetic interventions. PCD is divided into apoptotic type (apoptosis) and non-apoptotic type (such as ferroptosis, necroptosis, pyroptosis) (6–9). Apoptosis maintains cell membrane integrity and occurs in a caspase-dependent manner. In contrast, the main characteristics of non-apoptotic cell death are membrane rupture and caspase independence. PCD is a necessary event in multi-cellular organisms to maintain homeostasis by eliminating unrequired or damaged cells. However, some types of cell death, for example necroptosis, pyroptosis and ferroptosis, even apoptosis trigger an inflammatory response by releasing damage-associated molecular patterns (DAMPs), including HMGB1, IL-1α and IL-6, both of which are involved in the development of different kinds of inflammatory diseases, such as diabetes, Alzheimer’s disease, cancer. and even COVID-19 (10–15). Therefore, it is necessary to strictly control the inflammatory activation secondary to cell death and further explore the comprehensive mechanism of its activation.

Phosphorylation and dephosphorylation are the most common and widely studied post-translational modifications in eukaryotes (16). Reversible protein phosphorylation occurs mainly at serine, threonine and tyrosine and is mediated by kinases and phosphatases. Protein phosphorylation and dephosphorylation have been shown to effectively modulate immune responses. In recent years, increasing studies suggest that phosphorylation plays a critical role in regulating inflammatory cell death. For example, MK2 prevent TNF-induced apoptosis and necroptosis by directly phosphorylation of RIPK1 S321 residue (17). TBK1 and IKKϵ were also found to inhibit TNF-induced cell death *via* inducing the kinase RIPK1 phosphorylation (18). In addition, the kinases IKKα/β, TAK1, PLK1 and AMPK, and even the phosphatases Ppm1b and PTEN were shown to regulate inflammatory cell death through phosphorylation and dephosphorylation (19–24). In this review, we have outlined inflammatory cell death and phosphorylation, including apoptosis, pyroptosis, necroptosis and ferroptosis (**Table 1**). Moreover, we discuss the potential treatment strategy for diseases associated with cell death by targeting related protein kinases that mediate phosphorylation or phosphatases that mediate dephosphorylation.

**Table 1:** Comparison of the main features of different cell death.

Type	Apoptosis	Necroptosis	Pyroptosis	Ferroptosis
Morphological features	Cell membrane integrity, cell shrinkage, nuclear fragmentation, chromosomal DNA fragmentation	Cell swelling, nuclear chromatin concentration, nuclear fragmentation, plasma membrane bubbling and release of cellular contents	Cell swelling, plasma-membrane rupture, Chromatin condenses, nucleus remains intact, DNA random degradation	Lipid peroxides accumulation, mitochondrial membrane rupture and condense, mitochondria cristae loss
Initiators	Cell stress, radiation, DNA damage, growth factor withdrawal, mitotic defects, and hypoxia for intrinsic apoptosis; ligands of transmembrane receptor, such as TNFR1, FAS, DR4, and DR5 for extrinsic apoptosis	Ligands of TNFR1, FAS, TLR3, TLR4, and ZBP1 in the absence of active caspase-8	DAMPs and PAMPs	Uptake of cysteine is inhibited or the GPX4-dependent antioxidant is blocked

## Cell Death and Inflammation

Cell death can promote inflammation by releasing so-called endogenous DAMPs that promote the activation of dendritic cells, macrophages and other sentinel cells of the innate immune system (25). Excessive cell death can also directly and indirectly trigger an inflammatory response, leading to persistent inflammation (26).

Inflammatory activation signaling pathways is one of the pioneer innate immune responses. Acute inflammation is a defense response, usually caused by detecting extracellular stimulation and mediated by cytokines, chemokines, and other second messengers. Priming signals mainly consist of DAMPs and pathogen-associated molecular patterns (PAMPs), which activate cellular receptors on or within individual cells in exposed tissue (27). Thus, signaling molecules induce and regulate part and systemic inflammation, cell proliferation, and tissue repairmen (28). Cytokines amplify inflammation, increase the production of inflammatory mediators, coordinating part tissue and systemic immune responses.

Resolution of acute inflammation is critical to restoring homeostasis if danger or damage is handled. Failure to deal with the inflammatory response can lead to chronic inflammation. Amplifying the cycle to ensure adequate acute inflammatory responses, promote excessive inflammation and a long-lasting immune responses, leading to sustained tissue damage and ultimately to chronic inflammatory disease (29, 30).

During necroptosis, receptors downstream of TNF receptor 1 (TNFR1) interact, and activation of serine/threonine protein kinase 3 (RIPK3), which promotes the release of inflammatory cytokines and chemokines (31). Necroptosis causes early infiltration of the plasma membrane, and it is highly pro-inflammatory. Response to DAMPs activates pyroptosis, and sterile, while inflammation induced by sterile DAMPs is the underlying cause of inflammatory disease, which is activated after activation of pro-inflammatory caspases to a series of PAMPs and DAMPs (8, 32). Activated caspases can cleave a variety of substrates, including nucleases and gasdermin D, which promote DNA disruption, during both classical and non-classical pyroptosis processes. Once cracked, the N-terminal part of gasdermin D binds to the lipid membrane to forms oligomer pores. Due to increased osmotic pressure, cell swelling and cell membranes rupture, followed by the release of DAMPs, including ATP, HMGB1 and mitochondrial DNA (33, 34).

In classical pyroptosis processes, activated caspase 1 directly cleaves pro-IL-1β and pro-IL-18, and is released into the extracellular space through the pyroptoptic pores (35). Although the non-classical caspase cannot cleave pro-IL-1β and Pro-IL-18 directly, non-classical caspase cleavage of the gasdermin D can also lead to NLRP3 activation. Subsequent activation of caspase-1 depends on IL-1 β and IL-18 maturation (36).

Cell death plays an important role in cancer therapy, many anti-cancer therapies eliminate cancer cells by using modulate cell death pathways. Necroptosis can serve as an alternative mode of cell death that can potentially eliminate cancer cells and prevent tumorigenesis (37). Evasion of the apoptotic cell death program can facilitate tumor initiation and maintenance as well as promote therapeutic resistance (38).Pyroptosis has emerged as a key form of cancer that serve as a promising target for enhancing tumor immunity. It has been shown that the key pyroptotic proteins GSDMB and GSDME/DFNA5 can be cleaved by granzymes, especially granzymes A and B, respectively, to induce pyroptosis in cancer cells (39, 40). Triggering ferroptosis may prove to be a very effective means of selectively target and overcome iron-addicted and treat resistant tumor cells (41).

## Necroptosis and Phosphorylation

Necroptosis was previously thought to be accidental death due to cytotoxic over damage caused by a variety of routine molecular events (42, 43). But now necroptosis was thought the best characterized programmed and regulated cell death, including cell swelling, nuclear chromatin condensation, nuclear fragmentation, plasma membrane bubbling and release of cellular contents (1, 44).

The necroptosis pathway is initiated by numerous specific death receptors, including Z-nucleic acid binding protein 1 (ZBP1) (45), interferon receptors (IFNRs), tumor necrosis factor receptor (TNFR), and Toll-like receptors (TLRs) (46–48). The key molecules of necroptosis are receptor interacting protein kinase 1 (RIPK1), RIPK3, and mixed lineage kinase domain like pseudokinase(MLKL) (49). Many studies have shown that RIPK1 and RIPK3 form a RIP1-RIP3 complex (necrosome),which can be phosphorylated mutually (50, 51). MLKL oligomerize and translocate to plasma membrane, forming a RIPK1/RIPK3/MLKL complex to induce cell expansion and necroptosis (52, 53). MLKL is composed of N-terminal helical bundle domain (HBD) and a C-terminal kinase-like domain, and HBD is critical for the oligomerization of MLKL (54). MLKL oligomerization leads to dysfunction of plasma membrane ion channel, resulting in ion imbalance between internal and external compartments of the plasma membrane and ultimately leading to necroptosis (55). When caspase-8 is inhibited by the apoptosis inhibitor zVAD-fmk or other drugs, RIPK1 binds to RIPK3 to form necrosome. The kinase activity of RIPK3, the oligomerization of MLKL, and the formation of necrosome are considered to be important in the necroptosis processes (51, 53, 56).

Tumor necrosis factor receptor 1 (TNFR1) has been reported induces the expression of many genes that regulate inflammation in necroptosis (57). However, under certain conditions, during TNF-induced cell death, TNF-α is required to bind to TNFR1 on the cell membrane and recruit a range of proteins within the cell to form different complexes (58–60). Among them, complex I includes TNFR-associated death domain (TRADD), TRAF2, RIPK1, cylindromatosis, the cellular inhibitor of apoptosis protein 1 (cIAP1), and the ubiquitin complex (59).

The IκB kinase (IKK) complex consists of a nuclear factor-kappa B essential modulator (NEMO, also known as IKKγ) and two catalytic subunits (IKKα and IKKβ). It plays an important role in mediating immune inflammatory responses and promoting cell survival and tumorigenesis. NEMO recruits IKKα/IKKβ, leading to IKK-mediated rapid selective phosphorylation of IkBα. IkBα activates NF-kB and upregulates genes that encode pro-survival and pro-inflammatory molecules (61). IKKα/β is recruited by NEMO into complex I, which phosphorylates RIPK1 at Ser25 and inhibits RIPK1 kinase-dependent cell death (62, 63) (**Table 2**).

**Table 2:** Clinical Trials Targeting cell death -Related Kinases or Phosphatases.

Type	Phosphorylation Target	Pharmacological small molecules	Related diseases
**Apoptosis**	Caspase-8	Z-IETD-FMK, Berberine chloride	Atrophy, cancer, and neurodegenerative disease
	BAX/BAK	Kaempferol 3-O-rutinoside	
	RIPK1	SAR443122	Pulmonary diseases, neurodegenerative diseases, cardiovascular disease, Cutaneous Lupus Erythematosus, and cancer
	RIPK3	Dabrafenib, GSK872	
**Necroptosis**	MLKL	Necrosulfonamide	
	AMPK	Metformin	
	NLRP3	DFV890, ZYIL1	Sepsis, epilepsy, atherosclerosis, and Alzheimer’s disease, Primary Central Nervous System Lymphoma (PCNSL),Symptomatic Knee Osteoarthritis
**Pyroptosis**	BTK	DTRMWXHS-12, Ibrutinib, Zanubrutinib	
	AMPK	Metformin	Huntington’s disease, obstructive pulmonary disease, Alzheimer’s disease, Parkinson’s disease, Salivary Gland Carcinoma, Myelofibrosis
**Ferroptosis**	AKT	GSK690693, GSK2110183 (afuresertib), Ipatasertib	
	GSK3β	9-ING-41	

The complex II were consist of TRADD, RIPK1, FAS-associated death domain protein(FADD) and caspase 8 (64). Caspase 8 inactivates RIPK1 and RIPK3 through proteolytic cleavage. When caspase 8 is inhibited, the necrosome is formed, and RIPK1 and RIPK3 were phosphorylated and activated (65). MLKL mediates necroptosis by translocate to the plasma membrane and inducing rupture of plasma membrane. MLKL activation occurs in the necrosome, which is composed of MLKL, RIPK3 and RIPK1 (66, 67). In this necrosome complex, RIPK3 phosphorylates the activation ring of MLKL, promoting conformational changes and the formation of MLKL oligomers. It is found that the phosphorylation of Ser345 is important to RIPK3-mediated necroptosis, It is prove that Ser345 is not required to phosphorylate the interaction between RIPK3 and MLKL in the necrosome, but is critical for MLKL translocation, accumulation in the plasma membrane, and following necroptosis (68).

RIPK3 is an RHIM-domain kinase, it forms complexes with other RHIM-domain proteins, such as RIPK1, ZBP1, and TRIF under activation of necroptosis (67). RIPK3 is phosphorylated at many sites in the necrosome, after Ser227 of RIPK3 was autophosphorylation, it formed hydrogen bonds with Ser404 of MLKL, triggering MLKL to recruit necrosome, resulting in MLKL phosphorylation and induces necroptosis (51, 69). In addition to IKK and IKKβ, TANK-bound kinase 1 (TBK1) is recruited to complex I, which regulates RIPK1 phosphorylation (60, 70). TBK1/IKKϵ recruits complex I through the interactions with NEMO, NAP1, and TANK, then phosphorylated RIPK1 is following inhibited by RIPK1 kinase-dependent cell death. Compare to IKKα/β, TBK1/IKKϵ-mediated RIPK1 phosphorylation does not require TAK1 activation, suggesting that TBK1/IKKϵ phosphorylates and regulates RIPK1 in a manner independent of NF-κB signaling (60, 70). More recently, three separate experiments have proved that RIPK1-dependent cell death is controlled by phosphorylation of RIPK1-ser321 and Ser336 by MK2. In response to TNF signaling, TAK1 phosphorylates p38, which then activates MK2 (17, 71, 72). Active MK2 phosphorylates RIPK1 and it recruited by complex I, which inhibits the formation of complex II b by blocking the interaction of complex II b with FADD. Although TAK1 indirectly phosphorylates RIPK1 through activating IKKα/β and MK2, TAK1 has also been proved to directly phosphorylate RIPK1 (73). RIPK1 autophosphorylation leads to its enzymatic activation, which activates its cytotoxic function. It is shown that Ser14/15, Ser20, Ser161 and Ser166 were autophosphorylation sites in RIPK1 (74). Furthermore, knockdown of L929 cells by reconstructed RIPK1 in RIPK1 K45A/S161E or D138N/S161E mutants is enough to induce RIPK1-dependent necroptosis and the formation of necrosome. These results suggest that RIPK1 autophosphorylation at Ser161 is crucial in RIPK1-dependent cell death (75). RIPK3 is phosphorylated at many sites in the necrosome, in these sites, the autophosphorylation of RIPK3 on Ser227 bind with of the MLKL on Ser404 and formed the necrosome, which phosphorylates MLKL and induces necroptosis (51, 76). Ppm1b was separated by mass spectrometry as the binding partner of RIPK3, which dephosphorylated RIPK3 (77). Consistent with this result, Ppm1b KO mice showed higher mortality in TNF-induced SIRS and increased phosphorylation of RIPK3, suggesting that Ppm1b negatively regulates RIPK3 dephosphorylation under physiological conditions (20).

Parkin was identified as a RIPK3 E3 ligase (78). During the processes of necroptosis, AMP-activated protein kinase (AMPK) is activated by RIPKs and following phosphorylates Parkin’s Ser9. Phosphorylated Parkin causes polyubiquitination of the K33 link on Lys197, Lys302, and Lys364 of RIPK3, and inhibit the formation of necrosome (78). Mass spectrometry showed that MLKL and phosphoglycerate mutase family 5 (PGAM5) are downstream molecules of RIPK3 (79). The N-terminal of MLKL contains 4HB and the C-terminal contains a pseudokinase domain. In case of RIPK3 activation, MLKL is recruited into the necrosome through its interaction with RIPK3 and following phosphorylated on Thr357 and Ser358 in the pseudokinase domain. This phosphorylation of RIPK3 by MLKL leads to a conformational change in the 4HB domain, leading to oligomerization of MLKL (80–82).

MER receptor kinases (TAM), TYRO3 and AXL were identified as MLKL kinase. TAMs phosphorylate MLKL on Tyr376 in response to necroptosis stress and promotes MLKL oligomerization (83). The loss of TAMs attenuated necroptosis but did not alter the phosphorylation of RIPK1, RIPK3, or MLKL. It is suggesting that TAM phosphorylation of MLKL may be the final modification step of necroptosis.

Bcl-2 has been considered as a negative regulator of MLKL. Bcl-2 binds to MLKL and interferes with RIPK3-mediated MLKL phosphorylation and MLKL oligomerization (84). Beclin1 was considered as an inhibitory member of necrosome. Beclin1 binds to necrosome by interacting with MLKL during necroptosis. The interaction between Beclin1 and MLKL requires MLKL phosphorylation of RIPK3, suggesting that Beclin1 is the final barrier to necroptosiss (85).

**Figure 1:**
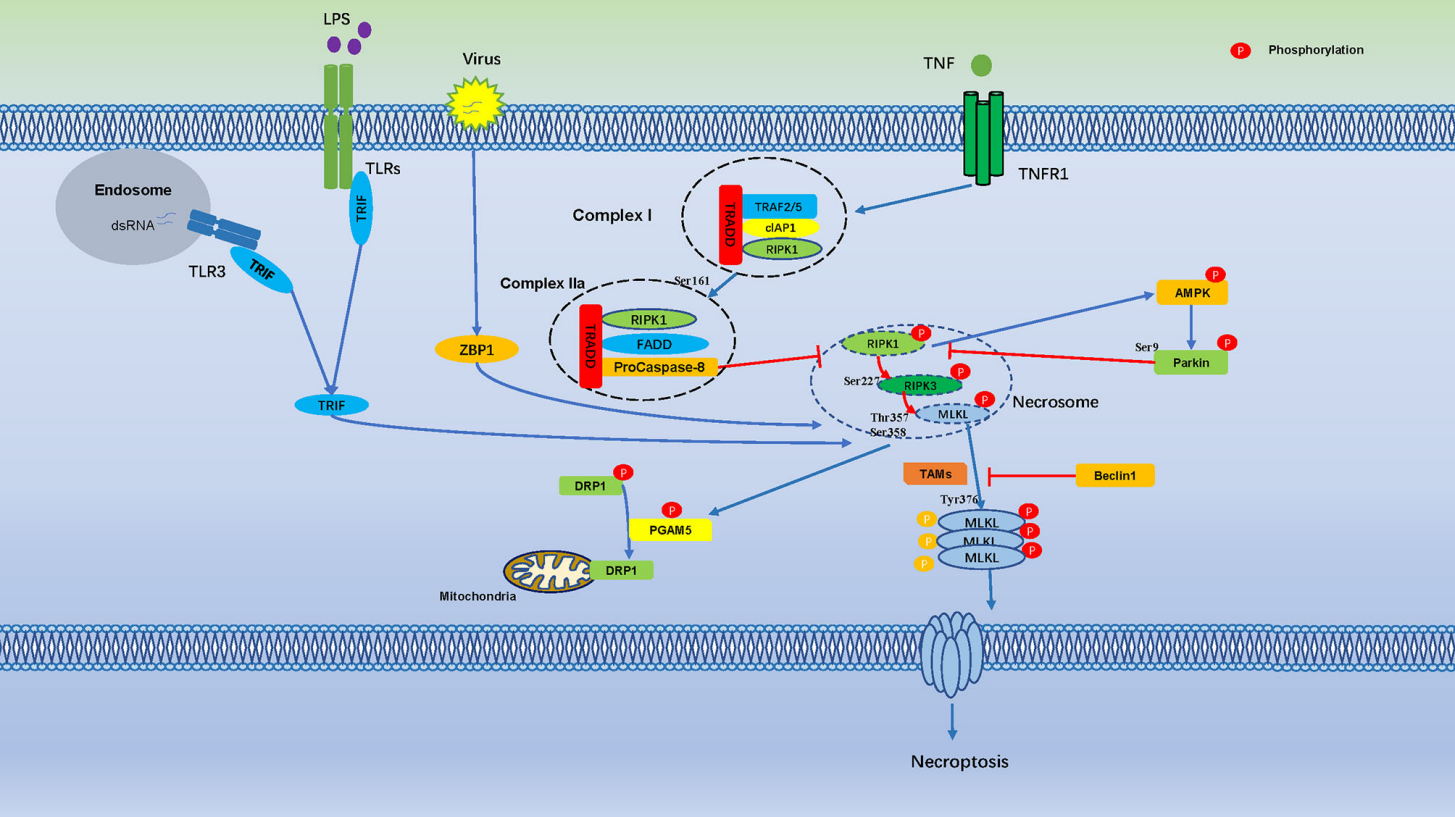
Cell Necroptosis is Regulated by Numerous Phosphorylation Events. Phosphorylation regulates cell necroptosis through numerous phosphorylation events, TNF bind to TNFR1 on the cell membrane and recruit a series of proteins to form complex I, which is includes TNFR-associated death domain (TRADD), RIPK1, TRAF2, the cellular inhibitor of apoptosis protein 1 (cIAP1). Autophosphorylation of RIPK1 leads to its enzymatic activation, Ser161 of RIPK1 is an important autophosphorylation sites, which triggers RIPK3 phosphorylation, the phosphorylation of Ser227 of RIPK3 triggers MLKL recruitment to the necrosome, which phosphorylates MLKL in Ser358, Thr357, and induces necroptosis. AMPK is activated by RIPKs and subsequently phosphorylates Parkin’s Ser9, which is inhibits phosphorylation of RIPK3. Necrosome promotes phosphorylation of PGAM5, which induces dephosphorylation of DRP1.

MLKL exhibits a similar phosphorylation dependence through RIPK3-dependent signaling prior to pore formation and subsequent necroptosis. Therefore, the phosphorylation of pore-forming proteins may be a common mechanism of cells regulate pore formation through death effector proteins (53) **(** **Figure 1** **)**.

## Apoptosis and Phosphorylation

Apoptosis is a process of cell suicide triggered by foreign factor, It’s the spontaneous, orderly death of cells controlled by certain genes, one highly regulated programmed cell death (PCD) (86), The characteristics of apoptosis are cell becoming small and fine cytoplasmic density increased, mitochondrial membrane permeability changed, cytochrome C released into the cytoplasm, the nucleus shrinks, and eventually forms apoptosome, which are then engulfed by phagocytes (87, 88). The regulation of apoptosis is mediated by two pathways: intrinsic and extrinsic apoptosis signaling pathways. Caspase plays the key role in many apoptotic regulatory pathways (46, 86, 89).

### The Intrinsic Pathway

The intrinsic pathway is caused by many of non-receptor-mediated stimuli, such as cellular stress, radiation, DNA damage, and hypoxia (90–92). In the presence of dATP, cytochrome C released into the cytoplasm and binds with apoptosis-related factors to form a polymer, and caspase-9 binds with it to form an apoptosome, when apoptosome activate, and then activates other caspases to induce apoptosis (93). After receiving these endogenous stimuli signals, the pro-apoptotic proteins BAX and BAK form pores on the mitochondrial membrane, destroy the mitochondrial membrane potential, and induce the release of the cytochrome C and apoptotic inducer into cytoplasm (90, 94). Cytochrome C binds to apoptotic peptidase activator 1 and pro-caspase-9, to form the apoptosome. The process of apoptosome formation is essential for the cleavage and activation of pro-caspase-9. Activated caspase-9 cleaves pro-caspase-3 and pro-caspase-7, activated caspase-3 and -7 ultimately initiate apoptosis (15, 95, 96).

### The Extrinsic Apoptosis Pathway

The extrinsic apoptosis pathway is triggered by many transmembrane receptors, such as Fas/FasL, death receptor 4 (DR4), death receptor 5 (DR5) and TNF-1/TNF-α, they are typical molecular model signals that caused to apoptosis (46, 97). Stimulated by ligands, these receptors recruit appropriate adaptors to synthesis of a protein complex called a death-inducing signaling complex (DISC) (98). These receptors and ligands activate procaspase by binding to the caspase-8/caspase-10 precursor protein through the Fas-associated DD protein (FADD), then FADD recruits pro-caspase-8 through the death effect domain and induces pro-Caspase-8 activation through its own proteolytic cleavage (68, 99). Activated caspase-8 cleaves and activates effector caspases, including caspase-3 and caspase-7, following activate the downstream effector molecules then leads to apoptosis (68, 99).

Apoptosis signals lead to activation of Mst through phosphorylation and proteolytic cleavage. Apoptotic stimuli result in Mst autophosphorylation and activate caspases. The autophosphorylated Mst is active, and phosphorylates substrates amplify the apoptosis signals, then increasing caspase activation. Futhermore, active caspases cleaved the regulatory domain of the Mst carboxy-terminal. This releases the active amino-terminal kinase domain that migrate to the nucleus, where they phosphorylate transcription factors and histones (100).

HSP27 inhibits ASK1 cell death signaling by phosphorylation of PKD1. After ischemia, PKD1 is phosphorylate, but without HSP27, unable to inhibit ASK1 activity and resulting in cell death (101).

Phosphorylation plays critical role in apoptosis, although the extent to which proteolysis and phosphorylation pathways interact during PCD remains unclear. Using a quantitative proteomic platform integrates phosphorylation sites into protein topologies, a set of over 500 apoptosis phosphorylation events has been identified and shown to be rich on cleaved proteins and clustered around caspase protein hydrolysis sites. Meanwhile, it was found that caspase-cleavage can expose new phosphorylation sites. Conversely, phosphorylation at the +3 position directly promote proteolysis of caspase-8 substrate. Tt is revealed a form of functional communication between phosphorylation and caspase proteolytic pathways, leading to increased protein cleavage rates and the emergence of new phosphorylation sites (102).

**Figure 2:**
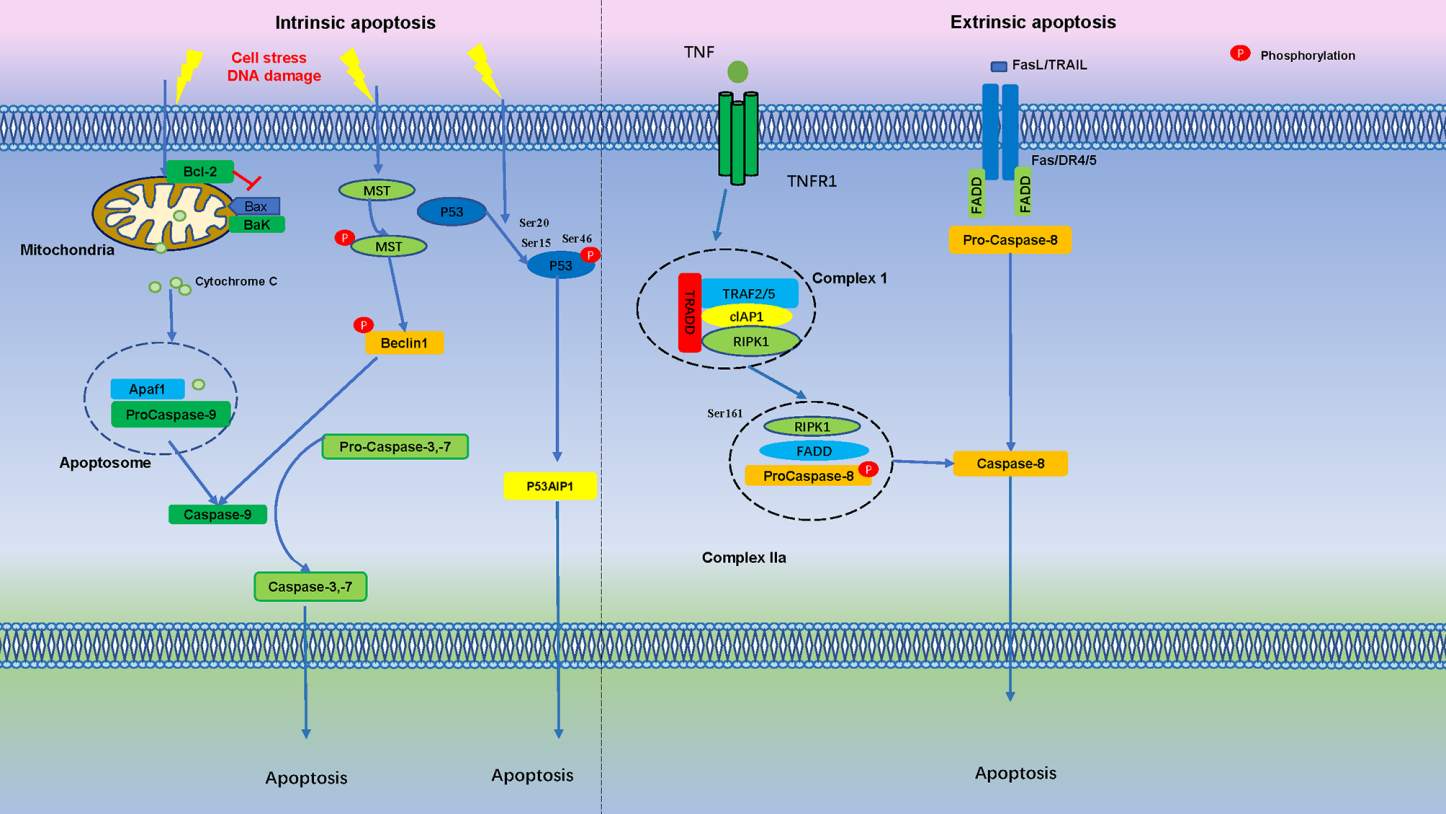
Apoptosis is Regulated by Numerous Phosphorylation Events. In the intrinsic apoptosis pathway, cell stress and DNA damage promotes mitochondria release cytochrome C into the cytoplasm, binds with Apaf1 and pro-caspase-9 to form an apoptosome. Apoptotic stimuli lead to MST autophosphorylation and triggers Beclin1 phosphorylation, which activate caspases. After initial DNA damage, p53 is phosphorylated at Ser15 and Ser20, if DNA damage is severe, phosphorylation of p53 protein at Ser-46 forms TP53INP1 and induce cell apoptosis. In the extrinsic apoptosis pathway, Fas/FasL, death receptor 4 (DR4), death receptor 5 (DR5) and TNFR1 bind to their ligands to induce pro-caspase-8 activation, which then cleaves and activates effector caspases, such as caspase-3 and caspase-7, further activation of downstream effector molecules then leads to apoptosis.

After DNA damage, p53 is phosphorylated on Ser-15 and Ser-20, stimulating p53 to bind to promoter regions of genes subset. If DNA damage is severe, TP53INP1 forms a protein complex with Ser-46 kinase HIPK2 and PKCδ, resulting in phosphorylation of p53 on Ser-46, and induction of p53AIP1 gene transcription and cell apoptosis (103). It is suggested that TP53INP1 induces phosphorylation of p53 protein on Ser-46 and apoptosis after DNA damage **(** **Figure 2** **)**.

## Pyroptosis and Phosphorylation

Pyroptosis is a newly discovered PCD in recent years. It is a main non-specific defense mechanism in the body, which plays an irreplaceable role in antagonizing the invasion of external pathogens and sensing the endogenous danger signals. In 2001, Cookson et al. (104) have found that there was a mode of death dependent on caspase-1 activity, which was different from apoptosis dependent on caspase-3 activity, and they defined cell pyroptosis as caspase-1 dependent cell death in the first time. Shao et al. have found that pyroptosis can be triggered by the activation of caspase-4/5/11 by intracellular LPS, and the activated caspase-4/5/11 ultimately induces pyroptosis through the cleavage of Gasdermin family proteins (105, 106). Therefore, they defined pyrotopsis as PCD mediated by the Gasdermin family.

Pyroptosis with some characteristics of cell necroptosis and apoptosis on the morphology. When cell pyroptosis occurs, the nucleus condensation, chromatin DNA random fracture degradation, Numerous pores appear in the cell membrane, Cell membranes lose their ability to regulate the movement of substances in and out, cell loss of ion balance, both inside and outside osmotic swelling and membrane rupture occurred, releasing active substances such as cell content, stimulate the body The immune response recruits more inflammatory cells and amplifies the inflammatory response (107, 108).When microorganisms infect host cells externally or internally, the pattern recognition receptor (PRR) located in the cytoplasm is associated with PAMP and DAMP recognize and bind to the corresponding ligand to assemble and form the intracellular multiprotein complex, which activates inflammatory caspase-1 and caspase-4/5/11 further cleavage of GSDMD protein punch holes on the cell membrane and promoted the occurrence of pyroptosis. At the same time, inflammasome acts on downstream molecules to promote the maturation of inflammatory cytokines (such as IL-1β, IL-18, IL-6, etc.), chemokines, adhesion molecules, and release them into the extracellular domain through the ruptured cell membrane, recruit and activate more inflammatory cells and amplify local and systemic inflammatory responses (109).The activation pathway of pyroptosis was divided into two groups: the classical cell pyroptosis pathway activated by inflammasome caspase-1 and the non-classical pyroptosis pathway activated by LPS.

### Classical Cell Pyroptosis Pathway

The activation of classical cell pyroptosis pathway mainly depends on PRRs receiving danger signal molecule stimulation, recruiting pro-caspase1 to assemble and form inflammasome through ASC, activating caspase-1 molecule to further cleaves downstream GSDMD protein and promoting pyroptosis (110).

### Non-Classical Pyroptosis Pathway

Unlike the classical pathway, the non-classical pathway depends on caspase-4/5/11 activation. In response to intracellular LPS stimulation, mouse caspase-11 and its human counterpart caspase-4/5 can bind directly to conserve structure lipid A of LPS and be activated. The activated caspase-4/5/11 further cleaves GSDMD protein and promotes cell pyroptosis (111, 112). GSDMD protein exists in cytoplasm and can be stimulated by caspase-1 activated in classical and caspase-4/5/11 activated in non-classical pyroptosis pathway. GSDMD protein is divided into lipophilic N-terminal domain and hydrophilic C-terminal domain at specific sites. They are connected by a joint region with a protease cleavage site (113). The N-terminal domain bind to the biofilm and aggregate in the inner side of the biofilm to form pores, where water molecules invade cells and trigger cell pyroptosis (114–116).

**Figure 3:**
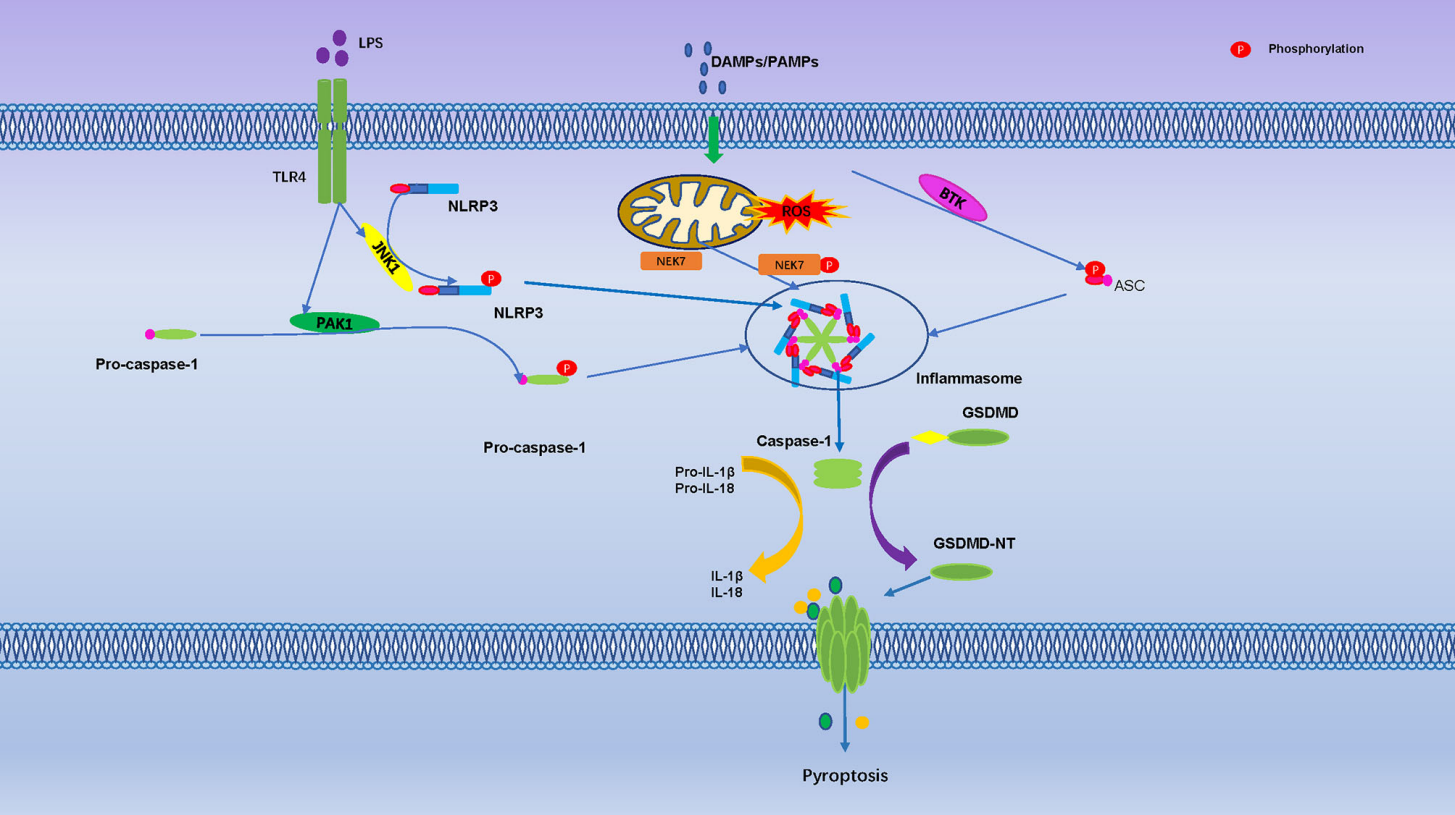
Pyroptosis is Regulated by Numerous Phosphorylation Events. The toll-like receptor 4 (TLR-4) activate Jun N terminal kinase 1(JNK1), which primes NLRP3 by direct phosphorylation at S198. The PAK1 promote phosphorylation of pro-caspase-1. Mitosis A-Related Kinase 7 (NEK7) and Bruton’s tyrosine kinase (BTK) promote NLRP3 activation through direct interaction. They are regulated activation of the NLRP3 inflammasome, cleavage of GSDMD protein punch holes on the cell membrane and promoted the occurrence of pyroptosis.

So far, evidence of phosphorylation only exists at Thr8 and Thr6 of human GSDMA and GSDME, both phosphorylation sites can prevent Gasdermin oligomerization and pore formation (117). Serine threonine kinase Polo-like kinase 1 (PLK1) mediates phosphorylation of GSDMA, but it is unclear if it phosphorylates Thr6 in GSDME (117, 118). GSDMD-NT, GSDMA-NT and GSDMA3-NT, can bind to membrane lipids only when phosphorylated, membrane lipid composition and phosphorylation modification were identified as another control mechanism for pyroptosis (119). In addition, TGF-β -activated kinase (TAK) 1 and PTEN has been found to control inflammasome activation and pyroptosis by regulating NLRP3 phosphorylation (19, 120) **(** **Figure 3**).

## Ferroptosis and Phosphorylation

It is have found that iron metabolism disorder and consequent iron overload are strongly linked to the occurrence and development of tumors. In addition, the presence of iron, especially iron divalent greatly aggravated the lipid peroxidation of saturated fatty acids (FAs) in human body. During n mitochondrial iron related oxidative phosphorylation, cells produce reactive oxygen species (ROS) and ATP. Overload ROS levels can lead to oxidative stress reactions that directly or indirectly destroy proteins, lipids and other macromolecules, leading to cell damage or death (121). Ferroptosis is recognized as non-apoptotic mediated cell death, usually caused by iron-dependent lipid peroxidation (122, 123). Ferroptosis characterized by the accumulation of lipid peroxides and lethal ROS, which is distinct from other types of cell death in morphologically, genetically, and biochemistry and is controlled by integrated oxidation and antioxidant systems. Morphologically, cells that undergo ferroptosis usually show typical necrotic morphological changes, such as cytoplasmic organelle swelling and rupture of plasma membrane, it is different from traditional apoptosis cells, which are characterized by blistering and contracting of plasma membrane, fragmentation and marginalization of chromatin, production of apoptosome, and cytoskeleton destruction (124).

Ferroptosis is an ROS-dependent form of cell death, associated with excess iron accumulation and lipid peroxidation, and changes in specific genes involved in regulating iron homeostasis and lipid peroxidation metabolism (125). Ferroptosis can be prevented by the use of iron chelators, while the provision of exogenous iron (such as ammonium ferric citrate) enhances ferroptosis (126). Glutathione-GPX4, FSP1-coenzyme Q10 (CoQ 10) and GTP cyclohydrolase-1- (GCH1-) tetrahydrobiotrexate (BH4) pathway have been confirmed to be involved in the regulation of ferroptosis (9, 127).

The phosphorylation typically occurs at the serine, tyrosine, and threonine residues of the target proteins (128). Energy stress causes cell death, which is related to the induction of reactive oxygen species (ROS) (129). Since lipid peroxidation is the main characteristic of ferroptosis, theoretically energy stress should promote ferroptosis. However, Lee et al. shown that glucose starvation inhibited erastin-induced ferroptosis in mouse embryonic fibroblasts, and this effect can also be observed in cystine depletion-, RSL3-, and GPX4-induced ferroptosis (129). AMPK is a key sensor of cellular energy status, and glucose starvation leads to phosphorylation and activation of AMPK (130). AMPK mediated the inhibitory effect of glucose starvation on ferroptosis by accelerating the phosphorylation of ACC and inhibiting lipid biosynthesis containing PUFA (129). Cancer cells with high AMPK are resistant to ferroptosis, and the inactivation of AMPK makes these cells sensitive to ferroptosis (129), it suggests that AMPK negatively regulates ferroptosis. During ferroptosis, BECN1 was identified as a novel SLC7A11/system Xc−binding protein that inhibit activity of type 1 ferroptosis inducers (such as erastin, sulfasalazine) (131, 132). Erastin and sulfasalazine activate AMPK as ferroptosis inducers, and AMPK phosphorylates BECN1 on Ser90/93/96, thereby promoting the formation of the BECN1-SLC7A11 complex (131). The interaction between BECN1 and SLC7A11 directly inhibit system Xc to promoting ferroptosis. Knockout of BECN1 could inhibited ferroptosis induced by system Xc inhibitors but not by RSL3, FIN56, or sulfoximine. Overexpression of BECN1 or application of the BECN1-activating peptide Tat-Beclin 1 promoted cancer cell ferroptosis (131). Although autophagy machinery are involved in ferroptosis, and BECN1 plays an important role in autophagy (133, 134). The downregulation of BECN1 cannot affect the formation of lipidated microtubule-associated protein 1 light chain 3 (MAP1LC3B) in ferroptosis (134). It suggests that, within the presence of certain stimuli, BECN1 bind to different partners to play different roles in autophagy and ferroptosis.

**Figure 4:**
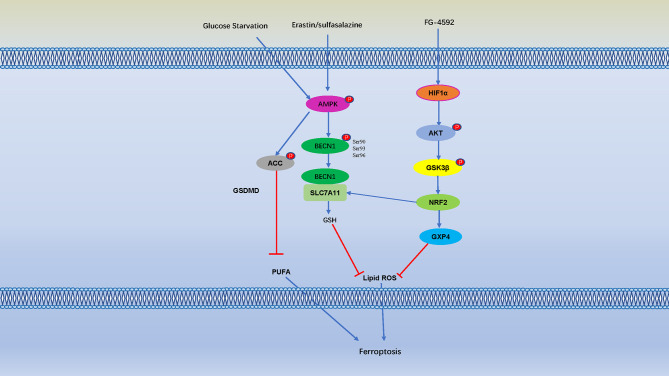
Cell Ferroptosis is Regulated by Numerous Phosphorylation Events. AMP-activated protein kinase (AMPK) is a key sensor of cellular energy status, and glucose starvation leads to phosphorylation and activation of AMPK, AMPK mediated phosphorylation of Acetyl-CoA carboxylase (ACC) and inhibiting PUFA-containing lipid biosynthesis and negatively regulates ferroptosis. Erastin and sulfasalazine also activate AMPK phosphorylation, and AMPK phosphorylates BECN1 at Ser90/93/96, thereby promoting the formation of the BECN1-SLC7A11 complex (131). The interaction between BECN1 and SLC7A11 promote GSH, thereby inhibit lipid ROS and negatively regulates ferroptosis. Moreover, folic acid induced kidney damage was alleviated by pretreatment of FG-4592, which increased phosphorylated of protein kinase B (AKT) and glycogen synthase kinase 3β (GSK-3 β) and activated NFE2-related factor 2 (Nrf2) to inhibit ferroptosis.

The Toll-like receptor 4- (TLR4-) nuclear factor kappa-light-chain-enhancer of activated B cells (NF-κB) signaling pathway activates the expression of multiple proinflammatory cytokine genes, and play an important role in inflammatory disorders (135, 136). Bone marrow-derived macrophages (BMDMs) pretreat with erastin obviously attenuates the expression of proinflammatory cytokines induced by LPS treatment, these effects are mediated by inhibition of the phosphorylation of IKKβ, and the phosphorylation and degradation of IκBα and NF-κB, consequently caused to inhibit the development of sepsis (136). Ferroptosis was detected in intestinal epithelial cells in patients with ulcerative colitis and colitis mice, it is indicate that NF-κBp65 phosphorylation suppresses ER stress-mediated ferroptosis in intestinal epithelial cells alleviates ulcerative colitis (137). Moreover, folic acid induced kidney damage was alleviated by FG-4592, which increased phosphorylated of AKT and GSK-3 β, and activated NFE2-related factor 2 (Nrf2) to inhibit ferroptosis (138). Protein kinase C (PKC) mediated phosphorylation of HSPB1 prevents erastin-induced ferroptosis by reducing lipid peroxidation (139) **(** **Figure 4** **)**.

## Targeting Phosphorylation for Treatment of Cell Death-Associated Inflammatory Disease

Protein kinases and phosphatases are highly regarded as very important drug targets, and now it is known that protein phosphorylation can be involved in regulating inflammatory cell death, which plays an important role in multiple diseases. Since many types of cell death are regulated by protein phosphorylation, targeted phosphorylation can be used to treat many inflammatory cell death-related diseases **(** **Table 2** **)**.

Necrosulfonamide is a very specific and potent inhibitor of MLKL, which inhibits MLKL-mediated necrosis by blocking its N-terminal CC domain. It inhibits necroptosis downstream of RIPK3 activation. Necrosulfonamide efficiently blocks necroptosis in human cells (140).

Metformin, as an AMPK activator, which inhibits the proliferation, migration, and activation of rheumatoid arthritis (RA)- fibroblast-like synoviocytes (FLS). Metformin treatment inhibited the expression of pro-inflammatory cytokines such as IL-6, TNF-α, and IL-1β, and increased the expression of p-AMPK-α1, protein kinase A α1 (PKA-α1), and hyaluronic acid and proteoglycan associated protein 1 (HAPLN1). The increased expression of HAPLN1 in RA-FLS may be beneficial for joint protection (141) (**Table 3**).

**Table 3:** Key kinases and phosphatases that regulate inflammatory cell death.

Types of cell death	Targets	Enzymes	Function and effect
Necroptosis	RIPK1	IKKα/β	Phosphorylation (–)
		TBK1	Phosphorylation (–)
		IKKϵ	Phosphorylation (–)
		MK2	Phosphorylation (–)
		TAK1	Phosphorylation (–)
	MLKL	RIPK3	Phosphorylation (+)
		TAM	Phosphorylation (+)
	RIPK3	Ppm1b	Dephosphorylation (–)
Apoptosis	P53	TP53INP1	Phosphorylation (+)
Pyroptosis	GSDMA	PLK1	Phosphorylation (–)
	NLRP3	TAK1	Phosphorylation (–)
		PTEN	Dephosphorylation (+)
Ferroptosis	BECN1	AMPK	Phosphorylation (+)
	HSPB1	PKC	Phosphorylation (–)

(–): Inhibit, (+): Promote.

## Concluding Remarks and Future Perspectives

**Figure 5:**
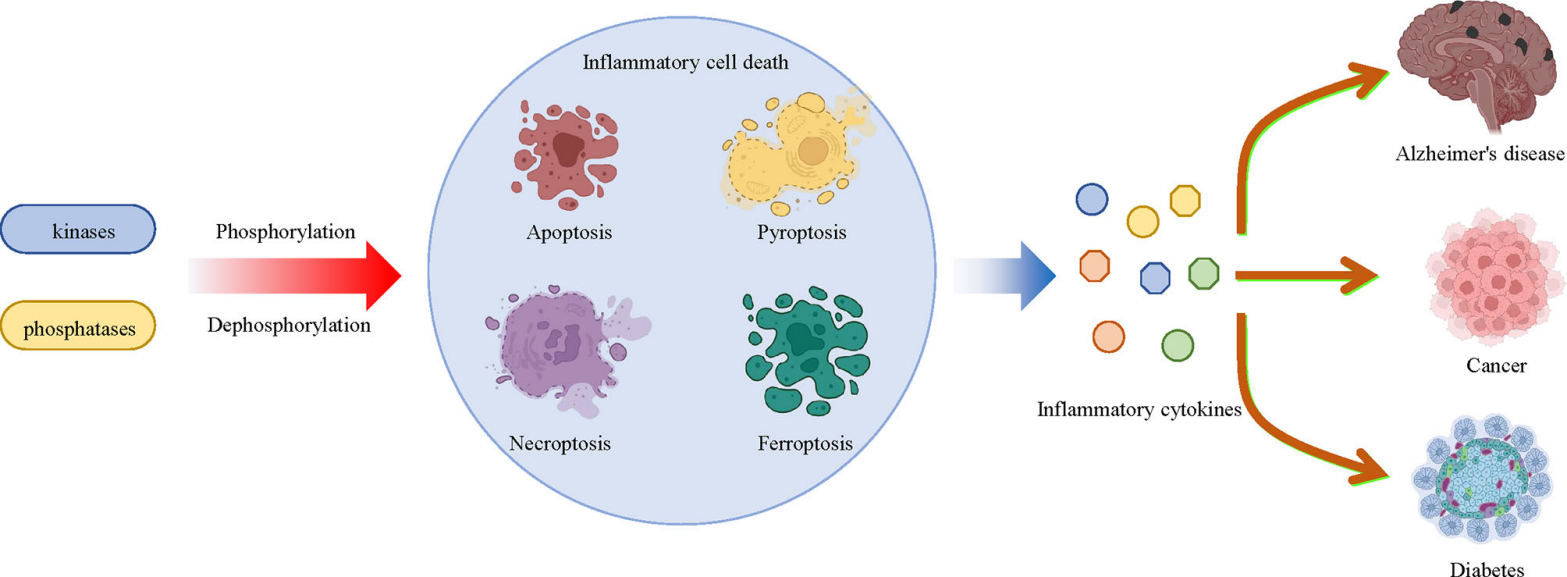
Multiple kinases and phosphatases regulate inflammatory cell death and related diseases through phosphorylation and dephosphorylation modifications. Multiple protein kinases that mediate phosphorylation and phosphatases that mediate dephosphorylation affect the pathologic development of a large number of inflammatory diseases, including diabetes, Alzheimer’s disease and cancer by regulating inflammatory cell death, such as apoptosis, pyroptosis, necroptosis and ferroptosis, and inflammatory cytokine release.

Cell death is a common process in all creatures, which is also involved in the development of various inflammatory diseases. Phosphorylation of proteins that regulate cell death is associated with a variety of inflammatory diseases, such as diabetes, Alzheimer’s disease, cancer, and so on, as mentioned above. In the past decade, there have been rapid advances in the understanding of how cell death regulates disease progression, including apoptosis, pyroptosis, necroptosis and ferroptosis, all of which are regulated by protein phosphorylation. Targeting the phosphorylation of MLKL, RIPK3, AMPK, BECN1 and other proteins can directly regulate cell death, which can then be used to treat cell death-related diseases by targeting these phosphorylated proteins. Understanding the relationship between inflammatory cell death and protein phosphorylation will not only improve our understanding of inflammation and cell death, but will also provide new targets and therapeutic strategies to inflammatory cell death-related diseases (**Figure 5**).

## Author Contributions

All authors listed have made a substantial, direct, and intellectual contribution to the work, and approved it for publication.

## Conflict of Interest

The authors declare that the research was conducted in the absence of any commercial or financial relationships that could be construed as a potential conflict of interest.

## Publisher’s Note

All claims expressed in this article are solely those of the authors and do not necessarily represent those of their affiliated organizations, or those of the publisher, the editors and the reviewers. Any product that may be evaluated in this article, or claim that may be made by its manufacturer, is not guaranteed or endorsed by the publisher.
